# The Association between Maternal Reproductive Age and Progression of Refractive Error in Urban Students in Beijing

**DOI:** 10.1371/journal.pone.0139383

**Published:** 2015-09-30

**Authors:** Zhong Lin, Guang Yun Mao, Balamurali Vasudevan, Zi Bing Jin, Kenneth J. Ciuffreda, Vishal Jhanji, Hong Jia Zhou, Ning Li Wang, Yuan Bo Liang

**Affiliations:** 1 The Eye Hospital, School of Ophthalmology and Optometry, Wenzhou Medical University, Wenzhou, Zhejiang, China; 2 School of Environmental Science & Public Health, Wenzhou Medical University, Wenzhou, Zhejiang, China; 3 College of Optometry, Mid Western University, Glendale, AZ, United States of America; 4 Department of Biological and Vision Sciences, SUNY College of Optometry, New York, NY, United States of America; 5 Department of Ophthalmology and Visual Sciences, The Chinese University of Hong Kong, Hong Kong, China; 6 Beijing Tongren Eye Center, Beijing Tongren Hospital, Capital Medical University; Beijing Ophthalmology & Visual Science Key Lab, Beijing, China; University of Florence, ITALY

## Abstract

**Purpose:**

To investigate the association between maternal reproductive age and their children’ refractive error progression in Chinese urban students.

**Methods:**

The Beijing Myopia Progression Study was a three-year cohort investigation. Cycloplegic refraction of these students at both baseline and follow-up vision examinations, as well as non-cycloplegic refraction of their parents at baseline, were performed. Student’s refractive change was defined as the cycloplegic spherical equivalent (SE) of the right eye at the final follow-up minus the cycloplegic SE of the right eye at baseline.

**Results:**

At the final follow-up, 241 students (62.4%) were reexamined. 226 students (58.5%) with completed refractive data, as well as completed parental reproductive age data, were enrolled. The average paternal and maternal age increased from 29.4 years and 27.5 years in 1993–1994 to 32.6 years and 29.2 years in 2003–2004, respectively. In the multivariate analysis, students who were younger (*β* = 0.08 diopter/year/year, P<0.001), with more myopic refraction at baseline (*β* = 0.02 diopter/year/diopter, P = 0.01), and with older maternal reproductive age (*β* = -0.18 diopter/year/decade, P = 0.01), had more myopic refractive change. After stratifying the parental reproductive age into quartile groups, children with older maternal reproductive age (trend test: P = 0.04) had more myopic refractive change, after adjusting for the children's age, baseline refraction, maternal refraction, and near work time. However, no significant association between myopic refractive change and paternal reproductive age was found.

**Conclusions:**

In this cohort, children with older maternal reproductive age had more myopic refractive change. This new risk factor for myopia progression may partially explain the faster myopic progression found in the Chinese population in recent decades.

## Introduction

Myopia is the most common visual disorder affecting children in East Asia [1–6]. Although there have been numerous studies, the precise etiology of myopia remains unclear. It is believed that both nature (genetic variation) and nurture (environmental variation) play important roles in myopic onset and its progression. On one hand, it was reported that myopia often exhibited strong familial clustering, in terms of correlations between myopia of the parents and their offspring, as well as between siblings [7–9]. In addition, several longitudinal studies have reported that the number of myopic parents was as a risk factor for the children’s myopic onset and/or myopic progression [10–14]. However, families share both their environment as well as genes. Environmental factors (e.g., near work and outdoor activity) were reported to be associated with myopic progression [15–17]. Recently, Liang et al. reported that the generational myopic shift from parents to their children was approximately 2 diopters (D) in an urban population of China[18], which was about 1 D higher than that found in a rural population[19]. These different magnitudes of generational myopic shift were attributed to different environmental exposures[18, 19].

There may be other more specific and subtle factors that have an effect on myopia. Using an epidemiological approach based on sequential “life stage” models, Rahi et al reported in a large British cohort (n = 2487) that increased maternal reproductive age was a putative risk factor, which was consistent with the global trend of increasing myopia[20]. In the same study, it was reported that greater maternal age (≥35 years) significantly increased the odds for the prevalence of myopia, myopic severity, and age of myopic onset in British adults[20]. For example, children with maternal age ≥35 years were 1.5 (95% CI 1.1–2.0) times more likely to have myopia, 2.3 (95% CI 1.1–4.7) times more likely to have high myopia, and 2.1 (95% CI 1.3–3.4) times more likely to develop myopia at an early age (<16 years) than children with maternal age 21–30 years[20]. Interestingly, children with older maternal age (>35 years) were 4.0 (95% CI 1.3–11.9) times more likely to have aniso-astigmatism per the Sydney Myopia Study[21]. Furthermore, and more general, it was well documented that either younger or advanced maternal age increased the risk of some congenital abnormalities, especially heart defects[22–24].

Mainland China has had a state policy of family planning for more than 30 years. The phenomenon of late marriage and late childbirth is extremely common, especially in the urban areas of China. Hence, it would be both interesting and important to ascertain the effect of maternal reproductive age on myopic refractive change among Chinese urban children. This effect may be helpful and be referred to when the macro family planning policy is developed in China. Thus, the aim of the present study, a subset of the Beijing Myopia Progression Study (BMPS)[25], was to investigate the association between maternal reproductive age and their children’s myopic refractive change.

## Methods

### Subjects

The study design, procedures, and baseline characteristics of BMPS were reported elsewhere[25]. Briefly, school children aged 6–17 years from Beijing were recruited from July to September, 2010. The inclusion criteria were: (1) best-corrected visual acuity (BCVA) 0.1 (log minimum angle of resolution, LogMAR) or better; and (2) willing to cooperate and return for scheduled annual visits. The exclusion criteria were: (1) presence of amblyopia and/or strabismus; (2) history of intraocular surgery or penetrating ocular trauma; and (3) serious medical/ocular health problems. The parents of these students were also invited to join the study. Then, the enrolled students were invited to be reexamined at the clinic center at a similar time of the year in 2011, 2012, and 2013. The vision examinations of the students included visual acuity, ocular biometry, cycloplegic refraction, and a detailed myopia-related questionnaire.

The study followed the tenets of the Declaration of Helsinki and was approved by the Beijing Tongren Hospital Ethics Committee. All participants (children and their parents) signed written informed assent/consent.

### Refractive Error

All students received a cycloplegic autorefraction (*Accuref-K9001*, *Shin Nippon*, *Japan*) at each vision examination, whereas the parents received a non-cycloplegic autorefraction (*Accuref-K9001*, *Shin Nippon*, *Japan*) only at the baseline vision examination. Three drops of cyclopentolate 1% (Cyclogyl, Alcon) were instilled in each eye. The second and third drops were instilled at 5 minutes and 15 minutes, respectively, after the initial instillation. Three readings were obtained in each eye and averaged in all participants. This autorefraction information was used to determine the distance refractive error.

### Definitions

Spherical equivalent (SE) refractive error was calculated as the sphere + 1/2 cylinder. The SE of the right and left eyes was highly correlated (Pearson correlation coefficient of the SE was 0.95, 0.96, and 0.93 for children, fathers, and mothers at baseline, respectively). Therefore, for simplicity, only data for the right eyes were used. Myopia, emmetropia, hyperopia, and high myopia were defined as SE < -0.5 diopters (D), -0.5D ≤ SE ≤0.5D, SE > 0.5D, and < -5.0D, respectively[26]. Children’s total refractive change was defined as the cycloplegic SE of the right eye at the final follow-up minus the cycloplegic SE of the right eye at baseline. The children’s mean annual refractive change was defined as the total refraction change divided by 3 (for the three year period of 2010 to 2013). Parental reproductive age was defined as the parental age minus their children’s age in years at baseline.

### Data Analysis

Population characteristics of the parents and children were represented as follows. Continuous variables such as children’s age and their baseline SE, etc. were described as the mean ± standard deviation (SD) when they were normally distributed, or represented with the median (lower quartile, upper quartile) if their distributions were skewed. Case (%) was used to describe the proportion of categorical variables.

Multiple general linear models (GLM) were used to evaluate the relationships (*β* and their 95% confidence interval [CI]) between the children’s refractive change and the putative risk factors. To extensively explore the association between the children’s refractive change and parental reproductive age (PRA), all models of the independent effects of PRA on the child’s refractive change were assessed in 2 ways: with PRA as a categorical variable (quartiles) and as a continuous variable (scaled to decade). Furthermore, the Loess procedure was used to intuitively display their relationships. To examine whether PRA was an independent risk factor for the offspring’s refractive change, the following potential confounders known to be associated with diopters based either on literature review or the results of stepwise screening were adjusted for, including the children’s gender, baseline refraction, paternal and maternal refraction, paternal and maternal reproductive age, paternal and maternal education years, near work, and outdoor activity time. When estimating the independent effects of PRA on the offspring’s refractive change, the lowest quartile of the PRA was selected as the reference group. All tests were two-sided, and p ≤0.05 was considered to be significant. Data management, figures, and all statistical analyses were performed using SAS 9.13 (SAS Institute Inc., Cary, NC, USA.). The relevant original data are within the S1 dataset.

## Results

For the 386 students at baseline, after adjusting for the children’s age, gender, paternal/maternal refraction, and time spent on near work and outdoor activity, no significant associations between either paternal or maternal reproductive age and the children’s refraction were found (*β* = 0.01 D/year, p = 0.95, and *β* = -0.01 D/year, p = 0.95). Similar results were found when using logistic analysis for the children’s myopia (SE< -0.5D) and high myopia (SE< -5.0D).

Of the baseline 386 students, 241 (62.4%) were reexamined in the final vision follow-up in 2013. Fifteen students were excluded as they refused cycloplegic refraction at the follow-up or had received orthokeratology after the baseline examination. Hence, 226 students (58.5%), including 110 boys (48.7%) and 116 (51.3%) girls with complete refractive data as well as complete parental reproductive age data, were enrolled for further analyses. The mean follow-up time and the mean annual refractive change were 35.6 ± 1.1 months and -0.49 ± 0.34 D/year, respectively, in these 226 students. The mean (range) paternal and maternal reproductive ages were 31.0 ± 4.0 (20.9–54.3) years and 28.4 ± 2.9 (19.0–36.8) years, respectively (Table 1). The children included in this current study, in general, were less myopic (-1.36 ± 2.42 D vs. -1.92 ± 2.53 D, p = 0.02), and with older paternal reproductive age (31.0 ± 4.0 years vs. 29.8 ± 4.0 years, p = 0.002) than in those that were excluded. No significant differences in the children’s age, gender, paternal/maternal refraction, paternal/maternal education years, near work and outdoor activity time were found between the included and excluded children. Both parents tended to have a child later, an average of 29.4 years and 27.5 years in 1993–1994 to 32.6 years and 29.2 years in 2003–2004, for paternal age and maternal age, respectively.

**Table 1:** Characteristics of children and their parents included in this study.

Variables	
**Number of children (male:female)**	110:111
**Baseline age (mean ± SD, years)**	
Children	10.7 ± 3.2
Father	41.8 ± 4.5
Mother	39.1 ± 4.0
**Baseline refraction (mean ± SD, diopter)** *	
Children	-1.36 ± 2.42
Father	-2.10 ± 2.56
Mother	-2.35 ± 2.54
**Children's annual refraction change (diopter/year)**	
Total	-0.49 ± 0.34
Male	-0.51 ± 0.34
Female	-0.46 ± 0.35
**Parental reproductive age (years)**	
Father	31.0 ± 4.0
Mother	28.4 ± 2.9

SD: standrad deviation

*Children’s baseline refraction was defined as the cycloplegic spherical equivalent (SE) of the right eye at baseline; parental baseline refraction was defined as the non-cycloplegic SE of the right eye at baseline.

In the univariate regression analysis with the children’s annual refractive change as the dependent variable, and the children’s age, gender, baseline refraction, paternal and maternal refraction, paternal and maternal reproductive age, paternal and maternal education years, near work, and outdoor time as the independent variable, respectively, the children’s age, baseline refraction, paternal and maternal refraction, and paternal and maternal reproductive age, were significantly associated with the children’s refractive change (Table 2). In the first multivariate regression model with the children’s annual refractive change as the dependent variable, and children’s age, gender, baseline refraction, paternal and maternal refraction, and paternal and maternal reproductive age as the independent variables, stepwise analysis showed that children who were younger (*β* = 0.07 D/year/year, p<0.001), with more myopic baseline refraction (*β* = 0.03 D/year/D, p = 0.002), and with older maternal reproductive age (*β* = -0.18 D/year/decade, p = 0.005) exhibited more annual myopic refractive change. In the second multivariate model with the children’s annual refractive change as the dependent variable, and adding the paternal and maternal education years, near work and outdoor activity time as the independent variables, and compared to model 1, the stepwise analysis revealed similar results for the children’s age (*β* = 0.08 D/year/year, p<0.001), baseline refraction (*β* = 0.02 D/year/D, p = 0.01), and maternal reproductive age (*β* = -0.18 D/year/decade, p = 0.01). However, no significant association between refractive change and paternal reproductive age was found with either model (Table 2). When adding the maternal gestational age and the children’s birth weight into the regression, no association between myopic refractive change and gestational age and birth weight was found (data not shown in Table 2).

**Table 2:** Associations between children’s refractive change (diopter/year) and putative risk factors in urban students in Beijing.

Parameters	Crude		Model 1			Model 2		
	*β* (95% CI)	P	*β* (95% CI)	P	VIF	*β* (95% CI)	P	VIF
**Age, years**	0.06 (0.05, 0.08)	<0.001	0.07 (0.06, 0.09)	<0.001	1.42	0.08 (0.06, 0.09)	<0.001	1.54
**Gender**	0.05 (-0.04, 0.14)	0.32						
**SE at baseline, diopter**	-0.02 (-0.04, 0.00)	0.05	0.03 (0.01, 0.05)	0.002	1.51	0.02 (0.00, 0.04)	0.01	1.50
**Paternal SE at baseline, diopter**	0.03 (0.01, 0.05)	0.002	0.01 (0.00, 0.03)	0.14	1.15	0.01 (0.00, 0.03)	0.10	1.17
**Maternal SE at baseline, diopter**	0.03 (0.01, 0.04)	0.006						
**Paternal reproductive age, decades**	-0.19 (-0.30, -0.08)	<0.001						
**Maternal reproductive age, decades**	-0.26 (-0.41, -0.10)	0.001	-0.18 (-0.31, -0.06)	0.005	1.02	-0.18 (-0.32, -0.05)	0.01	1.03
**Paternal education, years**	-0.01 (-0.02, 0.01)	0.35						
**Maternal education, years**	-0.01 (-0.03, 0.01)	0.26						
**Near work, hours/day**	0.02 (-0.01, 0.05)	0.12				-0.02 (-0.05, 0.00)	0.09	1.18
**Outdoor activity, hours/day**	0.00 (-0.04, 0.04)	0.92						

CI: confidence interval, VIF: variance inflation factor, SE: spherical equivalent. Model 1: the risk factors, including children’s age, gender, baseline SE, paternal and maternal SE, paternal and maternal reproductive age, were selected using the stepwise method; the parameters left in the final model were significant at the 0.15 level. Model 2: the risk factors of model 1 as well as paternal and maternal education years, near work and outdoor activity time, were selected using the stepwise method; the parameters left in the final model were significant at the 0.15 level.

**Fig 1:**
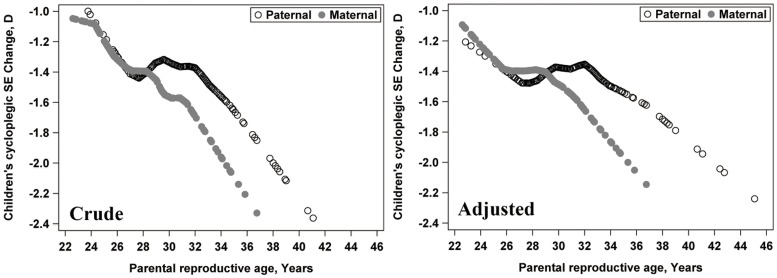
The relationship between the children’s cycloplegic spherical equivalent (SE) change and paternal/maternal reproductive age in all children using the multivariate LOESS regression model. Left, unadjusted; Right, father: adjusted for children's age, baseline cycloplegic SE, paternal non-cycloplegic SE, and near work time; mother: adjusted for children's age, baseline cycloplegic SE, maternal non-cycloplegic SE, and near work time.

Fig 1 presents both the unadjusted and adjusted (adjusted for children’s age, baseline cycloplegic refraction, paternal/maternal refraction, and near work time in all children) scatter plots between the children’s cycloplegic refractive change and paternal/maternal reproductive age. Both plots show a trend for the myopic shift to be greater with increased paternal reproductive age.

After dividing the parental reproductive age into quartile groups, children with older paternal reproductive age (from -0.44 ± 0.34 D/year to -0.58 ± 0.34 D/year) and maternal reproductive age (from -0.41 ± 0.31 D/year to -0.59 ± 0.36 D/year) had more annual myopic refractive change. In both multivariate regression models with the children’s annual refractive change as the dependent variable, and the children’s age, baseline refraction, paternal refraction, paternal/maternal reproductive age (model 1), and near work time (model 2) as the independent variables, children with the oldest maternal reproductive age (30.2–36.8 years) had significantly more annual myopic refractive change (*β* = -0.11 D/year/year, p = 0.03) compared to those with the youngest maternal reproductive age (19.0–26.4 years). Furthermore, children with older maternal reproductive age tended to have more annual myopic refractive change (trend test: p = 0.04) (Table 3). However, no significant trend for older paternal reproductive age and myopic refractive change (p = 0.35) was found.

**Table 3:** The association between parental reproductive age (years) and children's refractive change (diopter/year) in urban students in Beijing.

PRA, years	N	Mean ± SD	Crude		Model 1		Model 2	
			β (95% CI)	P	β (95% CI)	P	β (95% CI)	P
Father								
20.9~	56	-0.44 ± 0.34	Ref.	Ref.	Ref.	Ref.	Ref.	Ref.
28.2~	57	-0.45 ± 0.36	-0.01 (-0.13, 0.12)	0.91	0.04 (-0.06, 0.15)	0.42	0.03 (-0.07, 0.14)	0.54
30.8~	57	-0.48 ± 0.33	-0.04 (-0.16, 0.09)	0.57	0.00 (-0.10, 0.11)	0.98	0.00 (-0.10, 0.11)	0.96
33.2~54.3	56	-0.58 ± 0.34	-0.14 (-0.26, -0.01)	0.04	-0.02 (-0.13, 0.09)	0.69	-0.04 (-0.15, 0.07)	0.45
Trend				0.03		0.52		0.35
Mother								
19.0~	57	-0.41 ± 0.31	Ref.	Ref.	Ref.	Ref.	Ref.	Ref.
26.4~	55	-0.46 ± 0.36	-0.06 (-0.18, 0.07)	0.39	0.01 (-0.09, 0.12)	0.81	0.01 (-0.10, 0.11)	0.85
28.2~	58	-0.49 ± 0.33	-0.08 (-0.20, 0.05)	0.21	-0.03 (-0.13, 0.07)	0.57	-0.02 (-0.12, 0.09)	0.74
30.2~36.8	56	-0.59 ± 0.36	-0.18 (-0.31, -0.05)	0.01	-0.11 (-0.21, -0.01)	0.04	-0.11 (-0.22, -0.01)	0.03
Trend				0.01		0.03		0.04

PRA: parental reproductive age; SD: standard deviation; Ref.: reference group. Model 1: the risk factors, including children’s age, baseline SE, paternal SE, paternal/maternal reproductive age were adjusted. Model 2: the risk factors, including children’s age, baseline SE, paternal SE, paternal/maternal reproductive age, and near work time were adjusted.

## Discussion

The findings from the present longitudinal study suggested that students in urban Beijing with older maternal reproductive age were at risk for myopic refractive change. To the best of our knowledge, this is the first prospective study that has demonstrated an association between maternal reproductive age and myopic refractive change. In a retrospective cohort study, Rahi et al found that children with greater maternal age significantly increased the odds for the prevalence of myopia, myopic severity, and age of myopic onset in British adults[20]. In the present longitudinal study, Chinese children with older maternal reproductive age, especially more than 30 years, had more myopic refractive change. This is consistent with Rahi et al[20]. Furthermore, in the present sample of urban school children, the predicted myopic refractive change would be approximately 0.18 D greater per year for every 10 years increase in maternal reproductive age, after adjusting for crucial risk factors for myopia progression, such as age[5, 11, 13], baseline refraction[11], parental refractive error[10, 14], near work, and outdoor activity time[15–17], in the different multivariate models. However, unlike Rahi et al’s study[20], no association between reproductive age and either the children’s myopia or refractive error was found at baseline. This could be due to two reasons. First, the relatively small sample size in the present study may have undermined the outcome to some degree. Second, the present hospital-based study tended to enroll students with more myopic refraction (total median -1.44D[18] vs. -0.84D for 15-year-old girls in a suburb of Beijing[1]), since such students and their parents presumably may pay more attention to the child’s ocular and general health.

The findings from the present study, and that of Rahi et al, suggest prenatal “programming” or “patterning” on postnatal myopic development. The reasons for children with older maternal reproductive age, but not older paternal reproductive age, having more myopic refractive change remain somewhat elusive. However, cytoplasmic inheritance may be a factor. Certain inheritance material from the maternal cytoplasm may influence the children’s myopic development. Rahi et al reported that intrauterine growth retardation and smoking in early pregnancy were associated with the children’s myopia[20]. It was also reported that maternal drug misuse in utero was associated with a higher prevalence of strabismus and nystagmus, and further associated with long-term visual morbidity, such as lack of binocularity and poor visual acuity[27]. Thus, advanced maternal age and maternal drug misuse may have created different intrauterine environments for postnatal myopic development to occur. Other postnatal factors, such as postnatal light exposure, reported to be associated with myopia, should be considered[28]. Hence, studies dealing with myopic progression and detailed information on maternal pregnancy, delivery, and postnatal growth should be conducted.

Another interesting finding in the present study sample was a trend for an increasing number of births to older parents (approximately 1.7 years for mothers). It should be noted that all 226 children were the only child in their family. The increase in maternal reproductive age in this study was similar to a previous Taiwanese study, which enrolled women who delivered between the years 1990 and 2003 (also approximately 1.7 years as above) [29]. Children of older mothers were more likely to be premature, and thus to have lower birth weight[30]. Furthermore, shorter gestational age and lower birth weight were reported to be associated with more myopic refractive error at birth[31, 32]. Hence, older reproductive age may be thought of as the surrogate of less gestational age and lower birth weight. However, in the present study, no association between myopic refractive change and either gestational age or birth weight was found. It should be noted that the presence of either prematurity (<37 weeks) or lower birth weight (<2500g) was relatively low (5.4% and 2.8%, respectively) in the present sample. Furthermore, studies have reported lack of a relationship between myopia and either prematurity or low birth weight in kindergarten children born without retinopathy of prematurity or in adult twins[33–35]. Hence, it was unlike the association between maternal reproductive age and myopic refractive change via less gestational age or lower birth weight. Older reproductive age may also be thought of as the surrogate of either higher parental education or higher parental occupation/socioeconomic status. However, no association between myopic refractive change and parental education years, or parental occupation, was found in the present study. It should be noted that the maternal reproductive age was also likely to be associated with other unmeasured confounders, which might themselves be associated with the birth cohort. Hence, the children’s age was adjusted to compensate for this possible birth-cohort effect in this study, although it might not be fully compensate. Hence, larger confirmative studies enrolling children in a restricted age range to avoid unmeasured birth-cohort effects are warranted.

There were a few potential limitations in the present study. First, a relatively large proportion of the children were lost in the final follow-up. Since more primary school children were enrolled, this sample tended to be younger, less myopic, and with older paternal reproductive age (a tendency of older reproductive age in younger parents). Second, this hospital-based study tended to enroll children with more myopic refraction at baseline, as these parents presumably pay more attention to their child’s visual and ocular health. Third, due to the relatively small sample size, the range of maternal reproductive age was somewhat narrow, especially for the younger (<20 years, n = 1) and older (>35 years, n = 3) groups. Hence, school-based and population-based longitudinal studies with a larger sample size and wider maternal reproductive age are warranted.

In summary, urban students with older maternal reproductive age had more myopic refractive change. This new risk factor for myopic refractive change, as well as the increasing number of births to older mothers, may partially explain the faster myopic refractive change, especially in the urban areas of China, in recent decades. This new risk factor may be helpful when the macro government policy in China is developed.

## Supporting Information

S1 Dataset The relevant original dataset of this study. (XLS)
